# Association between Cigarette Smoking and Physical Fitness Level of Korean Adults and the Elderly

**DOI:** 10.3390/healthcare9020185

**Published:** 2021-02-09

**Authors:** Hyung Gyu Jeon, Gyuri Kim, Hee Seong Jeong, Wi-Young So

**Affiliations:** 1 Department of Physical Education, Yonsei University, Seoul 03722, Korea; hgjeon@yonsei.ac.kr (H.G.J.); 1965kmg@naver.com (G.K.); 2 Sports and Health Care Major, College of Humanities and Arts, Korea National University of Transportation, Chungju 27469, Korea

**Keywords:** physical fitness, physical measurement, smoking, tobacco

## Abstract

Although previous studies have examined the relationship between smoking and physical fitness, they only considered current smoking status and the same fitness measurements regardless of age. This study investigated differences in physical fitness based on tobacco smoking habits. A total of 2830 non-elderly adults (NEA; 19–64 years) and 629 elderly (65–89 years) participated in the study, using data extracted from a Korean national database. One-way ANCOVA and ANOVA were conducted to analyze the results. The subjects were classified into three groups (smokers, those who had quit, and never-smokers). In NEA men, a significant difference was observed in 50-m dash (*p* = 0.003) and 20-m shuttle-run (*p* < 0.001), while in elderly men differences were only seen in sit-ups (*p* = 0.015). In the case of NEA and elderly women, no significant differences were observed in physical fitness levels (*p* > 0.05). The decreased fitness level due to smoking was more noticeable in men than in women, and in NEA more than in elderly persons. A non-smoking policy and customized training based on age or gender are necessary to increase fitness and improve health conditions.

## 1. Introduction 

Consumption of tobacco is a major public health issue that has garnered significant importance in worldwide discussions. The World Health Organization and its partners run campaigns such as World No Tobacco Day to encourage people to quit smoking. This event is intended to promote smoking cessation and create awareness of the dangers of tobacco use and second-hand smoke exposure [1]. However, tobacco use remains a major public health concern, not only for elderly persons but also for the adult population, and is the cause of death for 50 percent of smokers, which amounts to approximately 7 million people worldwide each year [2,3,4]. Because tobacco smoke contains more than 4000 chemical components and additives, such as carcinogens and toxic heavy metals, inhalation of such smoke affects health by increasing the risk of various cancers [5,6]. Therefore, researchers need to focus on health problems caused by smoking.

Cigarette smoking not only has a direct negative impact on general health, but also has a negative impact on physical fitness level. Nikolakaros et al. [7] demonstrated that smoking is one predictor of decreased cardiopulmonary fitness. In addition, Su et al. [8] reported that current smoking was correlated with decreased physical fitness in tests of aerobic (3000-m run) and anaerobic (sit-ups and push-ups) capacity. Decreased physical ability stems from the fact that the increased levels of carbon monoxide caused by smoking reduce the amount of oxygen that is carried by hemoglobin. As smoking has a negative impact on vital organs, including the brain, nervous tissues, and the heart, which are essential for physical activity [9,10,11], all physical abilities can be affected. A previous study classified physical fitness into power, strength, endurance, cardiopulmonary endurance, flexibility, and speed [12]. Therefore, it is necessary to examine whether smoking affects various components of physical fitness.

A meta-analysis conducted by Bize et al. [13] reported that greater physical fitness has a consistently positive association with higher health-related quality of life. Individuals with high physical fitness are more functional, both in activities of daily life and in sports. However, since there are differences in physical characteristics between men and women, it is necessary to examine them separately. Further, in elderly adults, several studies have reported that poor balance, endurance, and upper-body and lower-body muscular strength are risk factors for falling. As falling can lead to fractures and even mortality, examining physical fitness among older adults is especially important [14,15]. Therefore, evaluating the influence of smoking on physical fitness by age and sex is an appropriate approach for understanding the relationship between health and quality of life.

Even after smoking cessation, the aforementioned toxic components and additives in tobacco could continue to affect physical health [16]. Decreased physical fitness associated with smoking is likely to not recover for years. Further exploration of smokers who quit would elucidate the acute and/or long-lasting effects of smoking on physical fitness level. Additionally, evidence that quitting smoking is associated with improved health-related quality of life and physical fitness could be used to reinforce policies and smokers’ willingness to quit smoking. However, it remains unclear whether smoking continues to affect physical fitness in those who have quit or whether quitting smoking can lead to improvement in various aspects of physical fitness.

Although previous studies have provided useful information regarding smoking and fitness levels by investigating the relationship between tobacco smoking and aerobic and anaerobic fitness levels [7,8], they have a few limitations. The major issue is that participants were generally classified based on their current smoking habits; past addictions were not taken into consideration. Furthermore, fitness levels in varied segments of the population were not considered. Few studies have examined the relationship between smoking and fitness levels in both non-elderly adults and elderly individuals. In addition, to the best of our knowledge, studies of the relationship between cigarettes and fitness levels for various activities, including aerobic and anaerobic fitness levels, in the Korean population have not been conducted. This study examined differences in physical fitness levels among non-elderly adults and elderly individuals according to different tobacco smoking habits, using information from a Korean database.

## 2. Materials and Methods

### 2.1. Participants

The 2015 Survey of National Physical Fitness was a cross-sectional epidemiological study conducted by the Institute of Sport Science and the Ministry of Culture, Sports, and Tourism in the Republic of Korea [17]. A total of 1204 non-elderly (men: *n* = 759, age = 38.23 ± 11.75, height = 172.27 ± 6.92, weight = 71.58 ± 8.35; women: *n* = 445, age = 39.43 ± 12.11, height = 159.19 ± 5.87, weight = 56.82 ± 6.89) and 251 elderly adults (men: *n* = 105, age = 72.69 ± 6.11, height = 162.85 ± 5.60, weight = 61.45 ± 7.01; women: *n* = 146, age = 74.29 ± 6.75, height = 150.80 ± 5.72, weight = 57.64 ± 8.15) were excluded from the current analysis due to missing data on smoking status or physical fitness measurements. Finally, participants consisted of 1683 non-elderly adult men and 1147 non-elderly adult women (a total of 2830 persons aged from 19–64 years), and 264 elderly men and 365 elderly women (a total of 629 persons aged between 65–89 years). Current smoking status was initially classified into three groups, based on responses to the following question: “Do you smoke cigarettes now?“ Possible answers were (1) I am a smoker (smokers); (2) I did, but not now (quitters); (3) I have never smoked (never-smokers). Among non-elderly adults, 511 were classified as smokers, 587 as having quit, and 1732 as never-smokers; elderly adults were classified as 47 smokers, 171 as having quit, and 411 as never-smokers. Information on age, sex, and physical fitness were additionally collected. To ensure representativeness of the data, samples were collected from nine states and included a mix of genders, ages, regions, and region sizes (large, small, and medium-sized cities, townships). Ethical approval from the institutional review board of Korea’s Institute of Sport Science and Ministry of Culture, Sports and Tourism was received. Informed consent was obtained from the participants. This study was conducted according to the principles in the Helsinki Declaration.

### 2.2. Measurement of Physical Fitness Level

Subjects participated in measurements of physical fitness after answering questions about their current smoking status. Well-trained researchers from the Korean Institute of Sport Science and the Department of Physical Education at a Korean university conducted the physical fitness measurements at local universities and fitness centers. The researchers were trained in the measurement procedures. Elderly individuals have different physical abilities compared to non-elderly adults and have a higher risk of injury during physical fitness tests. Accordingly, the tests conducted for the two groups were different. The physical fitness examination of non-elderly adults involved tests to determine grip strength (strength), sit-ups (endurance), standing long jump (power), 50-m dash (speed), sit-and-reach (flexibility), and a 20-m shuttle-run (cardiopulmonary endurance). The measurement items for the elderly persons included grip strength (strength), sit-ups (endurance—upper body), sit-to-stand (endurance—lower body), back scratch (flexibility upper body), sit-and-reach (flexibility—lower body), one-leg stand with eyes open (balance), and a 6-min walk (walking function). Detailed examination of the physical fitness measurement followed the procedure described in the ACSM’s Resource Manual for Guidelines for Exercise Testing and Prescription [17], the Advanced Fitness Assessment and Exercise Prescription [18], and the guidelines of the Senior Fitness Test Manual [19].

#### 2.2.1. 50 Meter Dash

We used the 50-m dash to evaluate speed as it has a high correlation with muscle power and agility and thus is useful as a basic fitness measurement item. A person positioned 3–5 m ahead of the start line gave the start signal and a 2 s delay was present between the preparation and start signal. The runners were required to be standing at the starting signal, and the seconds elapsed until the body of the participant touched the finish line were recorded. When measuring several people at once, the time for each person was recorded by each investigator. The 50-m dash measurement was conducted on the strait of a field track.

#### 2.2.2. 20 Meter Shuttle-Run

A 20-m shuttle-run was used to assess cardiopulmonary endurance of non-elderly adult participants. Cardiorespiratory endurance, which is useful for evaluating the functioning of the respiratory and circulatory systems during exercise, is an important component of basic physical fitness and is associated with the ability to continue complete body exercises for a sustained period of time. After the starting signal, the participant was required to cover the 20-m distance marked by plastic cones before the next signal was played. If the runner reached the mark before the signal, they were required to wait for the next signal. If the person did not reach the mark before the sound, another opportunity was provided, but if the runner failed to reach the mark again, the assessment was stopped. The number of times the 20-m mark was reached was recorded. One investigator was assigned to each runner.

#### 2.2.3. Sit-Ups

Sit-ups were used to evaluate muscular endurance, which is the ability to continuously utilize muscles for the same movement. The number of sit-ups per minute was counted. The participants were asked to lie on a rubber mat and ensure that the back was flat. They were instructed to keep the hands behind the head and flex the knees at an angle of 90 degrees while keeping the legs apart. The assistant then held each participant’s ankles and the individual had to raise the upper body and touch the elbows to the knees. This action was counted as one sit-up and was repeated continuously for the specified period of time.

#### 2.2.4. Grip Strength

The elderly can sustain injuries in the process of measuring strength. Therefore, in this study, grip strength was measured with minimal risk using a Smedley-type dynamometer (TTK-5401, Takei Scientific Instruments Co., Ltd., Tokyo, Japan) with 0.1 kg units. Participants were instructed to hold the dynamometer by wrapping it around the second node of the finger while standing. They were asked to keep the torso and arms at a 15-degree angle so that the dynamometer would not come in contact with the body. The investigators instructed participants not to swing the dynamometer or the body, so as not to involve other segment forces. Additionally, the arms and elbows had to be straight. This was performed twice by alternating from one side to the other; the highest strength reading was recorded.

#### 2.2.5. Standing Long Jump

The standing long jump was used to assess power, which implies exerting maximum force in a minimal amount of time. This was performed in a sandy field or a gym as it must not be practiced on concrete, and measurements were made in centimeters. Some caution is advised for a standing long-jump test. When the landing ground is sand, it should be maintained at the same height as the takeoff board, and in case of other ground materials, attention must be given to prevent injuries to the heel due to high impact force. The participant was required to stand in a comfortable position with feet on the takeoff board and jump as far as possible by bouncing the body without leaving the board. The distance of the heel landing point from the takeoff board was measured. The longest distance out of two attempts was recorded.

#### 2.2.6. Sit-And-Reach

Sit-and-reach was used to assess flexibility to determine the mean operating range of motion determined by the structure, muscular extensibility, and the tissues surrounding the joints. Measuring instruments and estimates in units of 0.1 cm were used. The participants were asked to sit barefoot so that the heel was completely in contact with the vertical plane of the measuring instrument, at which point the knees were required to be fully extended. The distance between each participant’s feet in this study was required to be less than 5 cm. Bouncing the upper body was prohibited in order to record accurate flexibility. The individual was asked to place both hands on the measurement ruler and then to push the measurement ruler straight with the fingers.

#### 2.2.7. Sit-To-Stand

Sit-to-stand is a test that was used to measure lower-extremity muscular endurance, which is primarily used for daily life functioning by the elderly. A 40 cm highchair was used for this test, and the number of times the participant could stand up perfectly in 30 s was measured. Individuals were asked to prepare by crossing their arms on their chest while standing and then to sit in the middle of the chair in the correct position. Before starting the test, their feet were required to touch the floor to prevent them from falling from the chair. Additionally, to prevent injury during the assessment, the chair was also fixed to the wall or the examiner held the chair. The examiner continued to pay attention to the subject’s condition; when participants reported discomfort in any body part, the test was stopped, and the examiner assisted participants to recover.

#### 2.2.8. Back Scratch

A frozen shoulder is a common pathological problem faced by the elderly that results in pain and limits one’s range of movement [20,21]. Back scratch is a test that is used to evaluate the flexibility of the upper body, including the shoulders. In a standing position, participants were asked to extend one hand with the palm facing down, from the shoulders to the back, and to reach as far as they could. Alternatively, with the palms facing up and the hands at the back of the waist, the participants were asked to extend the hands to touch or overlap the middle finger. After two practice rounds, two actual measurement trials were recorded and the highest value was recorded. A negative score was recorded if the middle fingers did not touch, a score of zero was recorded if the middle finger almost touched, and a positive score was assigned if the middle finger overlapped. Recordings were in centimeters.

#### 2.2.9. One-Leg Standing with Eyes Open

The one-leg standing position was used to evaluate postural control that is essential in daily life, such as when walking on flat and slippery ground, walking on uneven ground or crowded places, climbing stairs, and preventing falls. After one practice round, two actual trials were performed, and the highest score was recorded. The test continued until the foot touched the floor. The standing time was recorded at this point. During the examination, the investigators paid close attention to prevent the subjects from falling down.

#### 2.2.10. 6 Minutes Walking

Cardiovascular endurance assessment in elderly persons was conducted through a 6-min walking test because of the higher likelihood of physical limitations and health problems than in non-elderly adults [22,23]. The 6-min walk evaluates full-body endurance and the walking ability required to travel long distances. The elderly in general exhibit limited physical activity. The 6-min walking assessment is useful to estimate the physical activity level and volume. The elderly participants were instructed to walk as far as they could in 6 min on an oval field track. To give them an opportunity to adjust their pace, they were informed of the remaining time. After 6 min, participants were asked to stop. The distance traveled was assessed to the nearest 5-m mark. After the assessment, participants were instructed to slowly walk in place for a few minutes to cool down.

### 2.3. Statistical Analysis

The results of demographic characteristics and physical fitness levels are expressed as means and standard deviations. Since there are statistical differences in age for both non-elderly men and women, these were treated as covariates and incorporated in the analysis of the covariate model. One-way between-subjects analysis of variance was conducted to identify the mean difference between the smokers, those who quit smoking, and never-smokers for elderly adults. Statistical analyses were conducted using SPSS Statistics version 25.0 (IBM Corp., Armonk, NY, U.S.). When a group difference was identified, a Bonferroni-corrected post hoc test was also conducted. The alpha level for all analyses was set at 0.05.

## 3. Results

### 3.1. Demographic Characteristics of Participants

Descriptive demographic data of non-elderly and elderly adults are shown in Table 1. For non-elderly adult men, smoking status was associated with significant differences in age (F = 88.444, *p* < 0.001), height (F = 7.257, *p* < 0.001), and weight (F = 10.481, *p* < 0.001). Post hoc analyses revealed that the quit group was older than the smoker group (*p* < 0.001) and never-smoker group (*p* < 0.001), and smokers were older than never-smokers (*p* < 0.001). In non-elderly adult women, there was a significant difference in age (F = 4.606, *p* = 0.001) by group; post hoc analyses revealed that the never-smoker group was older than the smoker group (*p* = 0.037).

### 3.2. Differences in Physical Fitness Levels for Non-Elderly Adult Men

The results according to smoking habits and fitness levels among non-elderly adult men are shown in Table 2. In Korean non-elderly adult men, significant differences in fitness levels based on smoking status were observed for 50-m dash (F = 5.751, *p* = 0.003) and 20-m shuttle-run (F = 20.562, *p* < 0.001). Post hoc analyses revealed that the never-smoker group was faster than the smoker (*p* = 0.003) group. In the 20-m shuttle-run, the never-smoker group had greater running repetition than the smoker (*p* < 0.001) and quit groups (*p* = 0.006), and the smoker group had decreased running repetition compared to the smoker group (*p* < 0.001). There were no significant differences among the groups in grip strength, sit-ups, standing long jump, and sit-and-reach for non-elderly adult men (*p* > 0.05).

### 3.3. Differences in Physical Fitness Levels for Non-Elderly Adult Women

In non-elderly adult women, there were no statistically significant differences in fitness level based on smoking status (*p* > 0.05) for all fitness levels (Table 3).

### 3.4. Differences in Physical Fitness Levels for Elderly Men

Differences in fitness levels based on smoking status in elderly men are shown in Table 4. A significant difference was observed in the fitness level of elderly men for sit-ups (F = 4.253, *p* = 0.015). Post hoc analyses showed that the never-smoker (*p* = 0.014) and quit groups (*p* = 0.038) completed more repetitions of sit-ups than did the smoker group.

### 3.5. Differences in Physical Fitness Levels for Elderly Women

In elderly women, no statistically significant differences were observed, as for the non-elderly adult women (*p* > 0.05; Table 5).

## 4. Discussion

This study analyzed differences in physical fitness levels according to smoking habits of Korean non-elderly and elderly adults. There were significant differences in physical fitness levels among smokers, those who quit smoking, and never-smokers. These results support the findings of previous studies that smoking is a health-related risk factor and provide evidence that smoking can negatively affect physical fitness.

The study further demonstrates that differences exist in speed and cardiopulmonary endurance in Korean non-elderly adult men. A possible explanation for the decreased fitness associated with smoking could be impaired oxygen delivery, as habitual smoking decreases generation of adenosine triphosphate, such that there is a lack of energy to aid muscle contraction [23]. Additionally, another putative mechanism for the reduced physical fitness might be the negative effect of smoking on the circulatory system. Smoking can damage vascular endothelium and affect the respiratory muscle blood supply [24,25]. Our findings are consistent with Su et al. [8], who evaluated the association of smoking with aerobic and anaerobic fitness levels in Asian male soldiers. Their study concluded that smoking affects muscular and cardiovascular endurance, as evidenced by performance on push-ups, sit-ups, and long-distance running. The decreased muscular endurance observed in this study is consistent with previous research. Wüst et al. [26] demonstrated that smokers have lower fatigue resistance than never-smokers in the quadriceps muscle.

Additionally, smoking can negatively affect not only cardiopulmonary endurance, in which movements or cycles are repeated, but also speed, namely, instantaneous explosive performance. As smoking can increase proteolysis and inhibit protein synthesis, it could lead to loss of muscle mass [23]. Furthermore, the oxygen delivery system to the mitochondria and generation of adenosine triphosphate in mitochondria are impaired by smoking; these decreased abilities could affect muscular contraction and anaerobic fitness [23]. Moslemi-Haghighi et al. [27] and the present study demonstrated that the running speed of smokers was significantly slower than that of never-smokers. Additionally, similar to the results of our study, a previous study [28] that used an electric bicycle ergometer found a negative influence of smoking on lower-extremity power. Therefore, the results of the current study support previous studies that reported smoking is associated with decreased fitness.

The results of this study demonstrate that the quit group had greater muscular endurance than the smoker group in elderly men. These findings provide evidence that quitting smoking is beneficial to fitness and health. The results from a previous review provided significant evidence of positive effects of quitting smoking on the circulatory system, and pulmonary and cardiovascular function [29]. More specifically, forced expiratory volume in 1 s improved within 1 to 5 years in those who quit [30,31]. As these results denote improved lung function, this could be one possible explanation for the greater muscular endurance seen in those who have quit smoking.

However, not all physical fitness levels are affected by current smoking habits. There were no significant differences in strength, muscular endurance, power, and flexibility ability for non-elderly men. These findings are consistent with those of Jensen [32], who demonstrated no significant difference in muscular endurance between smokers and never-smokers. Another interesting observation is that cardiopulmonary endurance measured by a 2-mile running test was not associated with smoking [33]. Especially in women, the present study revealed no differences in all physical fitness measures, including aerobic and anaerobic, according to smoking status, regardless of age. However, as smoking increases the possible risk of women-specific diseases, such as breast cancer and cervical cancer, further studies may be required to determine women-specific physical fitness measurement with greater reliability and validity.

In general, the aging process decreases overall physical fitness, including strength, endurance, agility, and flexibility, and this leads to a less-active daily lifestyle. Although our data confirm that there were no significant differences in physical fitness levels for elderly people except muscular endurance in elderly men, the elderly have a high risk of diseases such as cardiovascular and respiratory disease [34]. Consequently, evaluating the fitness of the elderly population is very important as a first step toward preventing injuries and disease. Therefore, as the elderly population experiences aging effects, it is important that elderly persons quit smoking and improve their physical fitness to maintain high health-related quality of life.

This study indicates that the smoking population rate in women is lower than in men in both groups, that is, in non-elderly and elderly participants. These results differ from previous studies that investigated the smoking rate of the western population. Marcus et al. [35] reported that 22% to 26% of women smoked based on the geographical region in the United States. As women experience a higher number of symptoms and lower self-rated health as compared with men with similar smoking consumption patterns [36], the difference in smoking rate should be considered as an important indicator that may explain physical fitness differences among nations.

This study has strengths in that it suggests differences exist in various aspects of physical fitness according to current smoking status.

1. The present research was conducted using a Korean national database to demonstrate differences in the relationship between smoking habits and physical fitness in Asian individuals.
2. The fitness measurement tests performed by all participants were standardized and performed following a protocol proposed by Korea’s Sports Science Institute of the Ministry of Culture, Sports and Tourism.
3. Fitness measurement tests that took age into consideration were used to prevent injury and to generate accurate information.

However, the study also had limitations that must be recognized.

1. As the age range of the non-elderly adult category was quite wide, it is necessary to analyze this group further in detail. Although we controlled the age of participants of the non-elderly adults by treating it as a covariate, non-elderly adults would be defined in various ways, and elderly persons could be classified as middle-old age and superaged.
2. Although the 2015 Survey of National Physical Fitness that was collected by the Korean nation included not only physical fitness levels but also various information about health-related variables, there were some subjects who did not participate in smoking surveys or physical fitness measurements during the process of collecting information.
3. As the study design was cross-sectional, we could explain differences based on current smoking status, but we were unable to assess the cause-and-effect relationship between smoking and fitness level.
4. Although the present study used a large database, the sample size of quit and smoker groups in non-elderly and elderly women was insufficient to provide high reliability. However, it is of value to reveal that most Korean women do not smoke, and there could be associations among fitness levels and health issues related to smoking consumption among other nationalities.
5. The groups were classified as smokers, those who had quit, and never-smokers; however, information regarding smoking duration and frequency was not collected. Furthermore, there may be heavy and light smokers in the category that was generalized as smokers. Therefore, further research should carefully consider the potential impact of these factors.
6. Although gender, age, region, and region size of participants were considered when establishing the national database, there were significant differences in demographic characteristics in several groups.

## 5. Conclusions

Smoking can negatively affect aerobic and anaerobic fitness levels as well as health conditions. This study indicates that fitness may decrease more in men than in women and in the non-elderly than the elderly because of the impact of smoking. Therefore, it is necessary to encourage people to quit smoking and to introduce aggressive non-smoking policies. Furthermore, it would help to develop and disseminate customized training based on age group to increase fitness levels.

## Figures and Tables

**Table 1:** Demographics and physical characteristics of non-elderly adult and elderly participants.

**Variables**	**Non-Elderly Adults Men (*n* = 1683)**					**Non-Elderly Adults Women (*n* = 1147)**				
	**Smoker** **(*n* = 487)**	**Quit** **(*n* = 558)**	**Never Smoker** **(*n* = 638)**	***p***	**Post-Hoc**	**Smoker** **(*n* = 24)**	**Quit** **(*n* = 29)**	**Never Smoker** **(*n* = 1094)**	***p***	**Post-Hoc**
Age (years)	37.56 ± 11.74	43.37 ± 12.07	34.09 ± 12.38	<0.001	SG > NSG QG > SG,NSG	32.92 ± 11.58	35.31 ± 12.99	39.66 ± 13.09	0.010	NSG > SG
Height (cm)	173.76 ± 5.61	172.36 ± 6.49	173.26 ± 6.08	0.001	SG,NS > QG	159.84 ± 5.63	162.04 ± 6.27	159.98 ± 5.68	0.155	-
Weight (kg)	74.44 ± 9.87	73.51 ± 9.62	71.85 ± 9.53	<0.001	SG,QS > NS	55.62 ± 9.87	58.28 ± 7.85	56.99 ± 7.64	0.454	-
**Variables**	**Elderly Men (*n* = 264)**					**Elderly Women (*n* = 365)**				
	**Smoker** **(*n* = 39)**	**Quit** **(*n* = 144)**	**Never Smoker** **(*n* = 81)**	***p***	**Post-Hoc**	**Smoker** **(*n* = 8)**	**Quit** **(*n* = 27)**	**Never Smoker** **(*n* = 330)**	***p***	**Post-Hoc**
Age (years)	73.67 ± 4.86	71.99 ± 5.58	71.88 ± 4.73	0.166	-	76.88 ± 6.42	73.74 ± 6.37	73.32 ± 5.79	0.227	-
Height (cm)	166.35 ± 5.06	166.23 ± 5.51	164.73 ± 5.66	0.116	-	155.9 ± 6.63	154.66 ± 5.93	153.74 ± 5.43	0.400	-
Weight (kg)	65.98 ± 7.49	66.60 ± 7.76	66.17 ± 8.63	0.878	-	57.86 ± 8.64	57.71 ± 5.31	56.55 ± 7.31	0.646	-

Results are expressed as mean ± standard deviation. NSG: never-smoker group; QG: quit group; SG: smoker group.

**Table 2:** Differences in physical fitness variables based on smoking in non-elderly adult men.

Variables		Overall (*n* = 1,683)	Smoker (*n* = 487)	Quit (*n* = 558)	Never Smoker (*n* = 638)	*F*	*p*	Post-Hoc
Strength	Grip strength (kg)	42.75 ± 9.09	43.24 ± 9.70	42.36 ± 8.70	42.72 ± 8.94	1.162	0.200	-
ME	Sit-ups (reps/60 s)	34.75 ± 11.94	34.3 ± 12.45	32.49 ± 11.29	37.08 ± 11.68	1.895	0.151	-
Power	Standing long jump (cm)	197.71 ± 31.59	199.60 ± 30.88	190.95 ± 30.87	202.18 ± 31.79	0.680	0.507	-
Speed	50-m dash (s)	8.89 ± 1.69	9.04 ± 1.77	9.20 ± 1.52	8.51 ± 1.71	5.751	0.003	SG > NSG
Flexibility	Sit-and-reach (cm)	9.37 ± 8.42	9.21 ± 7.92	9.00 ± 8.43	9.82 ± 8.76	0.423	0.655	-
CE	20-m shuttle-run (reps)	32.87 ± 17.15	29.82 ± 15.21	30.11 ± 15.56	37.61 ± 18.77	20.562	<0.001	NSG > SG,QG QG > SG

Results are expressed as mean ± standard deviation. CE: cardiopulmonary endurance; ME: muscular endurance; NSG: never-smoker group; QG: quit group; SG: smoker group.

**Table 3:** Differences in physical fitness variables based on smoking in non-elderly adult women.

Variables		Overall (*n* = 1,147)	Smoker (*n* = 24)	Quit (*n* = 29)	Never smoker (*n* = 1,094)	*F*	*p*	Post-hoc
Strength	Grip strength (kg)	25.01 ± 5.99	24.28 ± 4.32	23.47 ± 6.51	25.07 ± 6.00	1.154	0.215	-
ME	Sit-ups (reps/60 s)	21.01 ± 12.37	22.92 ± 15.57	21.21 ± 11.78	20.96 ± 12.31	0.585	0.557	-
Power	Standing long jump (cm)	139.87 ± 26.26	148.25 ± 24.31	140.52 ± 28.69	139.67 ± 26.23	0.382	0.682	-
Speed	50-m dash (s)	11.34 ± 2.00	11.42 ± 2.79	11.25 ± 1.63	11.34 ± 1.99	1.969	0.140	-
Flexibility	Sit-and-reach (cm)	14.71 ± 8.25	13.48 ± 8.31	12.50 ± 8.90	14.79 ± 8.23	1.235	0.291	-
CE	20-m shuttle-run (reps)	18.15 ± 8.26	16.42 ± 6.87	19.00 ± 8.27	18.16 ± 8.29	2.732	0.066	-

Results are expressed as mean ± standard deviation. ME: muscular endurance; CE: cardiopulmonary endurance.

**Table 4:** Differences in physical fitness variables based on smoking in elderly men.

Variables		Overall (*n* = 264)	Smoker (*n* = 39)	Quit (*n* = 144)	Never Smoker (*n* = 81)	*F*	*p*	Post-Hoc
Strength	Grip strength (kg)	32.14 ± 7.69	30.72 ± 6.96	32.02 ± 7.55	33.03 ± 8.24	1.231	0.294	-
ME	Sit-ups (reps/60 s)	13.3 ± 9.98	9.18 ± 6.55	13.65 ± 9.80	14.64 ± 11.17	4.253	0.015	NSG,QG > SG
	Sit-to-stand (reps/30 s)	19.31 ± 7.75	16.82 ± 7.18	19.81 ± 7.91	19.62 ± 7.58	2.396	0.093	-
Flexibility	Back scratch (cm)	−9.51 ± 12.32	−7.08 ± 14.83	−8.88 ± 12.30	−11.78 ± 10.73	2.352	0.097	-
	Sit-and-reach (cm)	4.30 ± 9.01	2.84 ± 8.88	3.65 ± 8.74	6.16 ± 9.36	2.648	0.073	-
Balance	One-leg standing test (s)	33.23 ± 35.19	29.12 ± 32.02	33.16 ± 33.57	35.31 ± 39.44	0.407	0.666	-
CE	6-min walk (m)	553.5 ± 123.9	519.13 ± 117.91	557.64 ± 120.79	562.68 ± 130.74	1.814	0.165	-

Results are expressed as mean ± standard deviation. CE: cardiopulmonary endurance; ME: muscular endurance; NSG: never-smoker group; QG: quit group; SG: smoker group.

**Table 5:** Differences in physical fitness variables based on smoking in elderly women.

Variables		Overall (*n* = 365)	Smoker (*n* = 8)	Quit (*n* = 27)	Never Smoker (*n* = 330)	*F*	*p*	Post-Hoc
Strength	Grip strength (kg)	20.48 ± 5.40	20.18 ± 4.87	22.04 ± 4.03	20.36 ± 5.50	1.226	0.295	-
ME	Sit-ups (reps/60 s)	5.32 ± 7.44	5.88 ± 6.29	6.85 ± 7.79	5.18 ± 7.44	0.650	0.523	-
	Sit-to-stand (reps/30 s)	16.22 ± 6.42	12.00 ± 7.25	15.41 ± 5.73	16.39 ± 6.43	2.076	0.127	-
Flexibility	Back scratch (cm)	−3.90 ± 10.13	−1.04 ± 11.41	−5.08 ± 10.97	−3.87 ± 10.04	0.503	0.605	-
	Sit-and-reach (cm)	10.61 ± 8.58	14.21 ± 11.80	10.56 ± 9.72	10.53 ± 8.41	0.720	0.488	-
Balance	One-leg standing test (s)	22.35 ± 25.91	37.48 ± 37.86	19.90 ± 21.58	22.18 ± 25.88	1.494	0.226	-
CE	6-min walk (m)	485.68 ± 135.59	429.63 ± 46.51	476.78 ± 181.55	487.77 ± 132.67	0.780	0.459	-

Results are expressed as mean ± standard deviation. ME: muscular endurance; CE: cardiopulmonary endurance.

## Data Availability

The data that support the findings of this study are openly available in Institute of Sport Science and the Ministry of Culture, Sports, and Tourism at https://www.sports.re.kr.
